# The simultaneous measurement of quaternary mixture in over-the-counter cold medications using sequential spectrophotometric resolution approach enhanced with in-lab sample enrichment

**DOI:** 10.1186/s13065-023-00931-4

**Published:** 2023-03-22

**Authors:** Khadiga M. Kelani, Mohamed S. Emara, Ahmed W. Madkour, Hany A. Batakoushy, Rehab M. Tony

**Affiliations:** 1 Analytical Chemistry Department, Faculty of Pharmacy, Cairo University, El-Kasr El-Aini Street, PO 11562, Cairo, Egypt; 2 Pharmaceutical Analytical Chemistry Department, Faculty of Pharmacy, Al-Azhar University, Nasr City, Cairo, 11751 Egypt; 3 Pharmaceutical Analytical Chemistry Department, Faculty of Pharmacy, Menoufia University, Shebin Elkom, 32511 Egypt; 4 Pharmaceutical Analytical Chemistry Department, Faculty of Pharmacy, Modern University for Technology and Information, Cairo, Egypt

**Keywords:** Caffeine, Pseudoephedrine hydrochloride, Doxylamine succinate, Paracetamol, Successive ratio subtraction, Constant multiplication

## Abstract

A sequential spectrophotometric resolution technique (SSRT) was developed in this study without the use of systematic separation procedures to determine drug of a quaternary combination; caffeine (CAF), pseudoephedrine (PSE), doxylamine succinate (DOX), and paracetamol (PAR). Their presence in a tablet with a gap ratio of 3:3:1:150, respectively, and their overlapping spectra with low absorptivities make their resolution and determination impossible without prior separation. successive ratio subtraction technique (SRST) and constant multiplication method were used to solve these problems. Furthermore, an in-lab sample enrichment technique was applied to increase minor components concentration and consequently their absorbanses (CAF, PSE, and DOX). The D^0^ absorption spectra were generated by successive ratios followed by subtraction and multiplication of the constants. The maximum absorbances of the drugs tested, namely (CAF, PSE, DOX and PAR) were measured at wavelengths of 272.0, 257.0, 260.0, and 248.0 nm, respectively. The limits of detection (LOD) and limits of quantification (LOQ) were 0.021, 0.124, 0.186, 0.137 and 0.070, 0.414, 0.621, 0.456 (µg/mL), respectively. The linearitiy ranges (µg/mL) were 1.0–22.0, 1.0–24.0, 10.0–90.0 and 1.0–15.0 for CAF, PSE, DOX, and PAR, respectively. The International Conference on Harmonization (ICH) guidelines were applied for method validation, and the results obtained were within the limited parameters. The finding results were compared to official and/or published analytical methods to determine the procedure's reliability. It was noted that there was no actual difference in accuracy and precision between both meyhods. The proposed technique is sensitive, selective and economic;so it can be applied to the simultaneous analysis of these drugs in their commercial tablets and/or in quality-control laboratories.

## Introduction

**Fig. 1:**
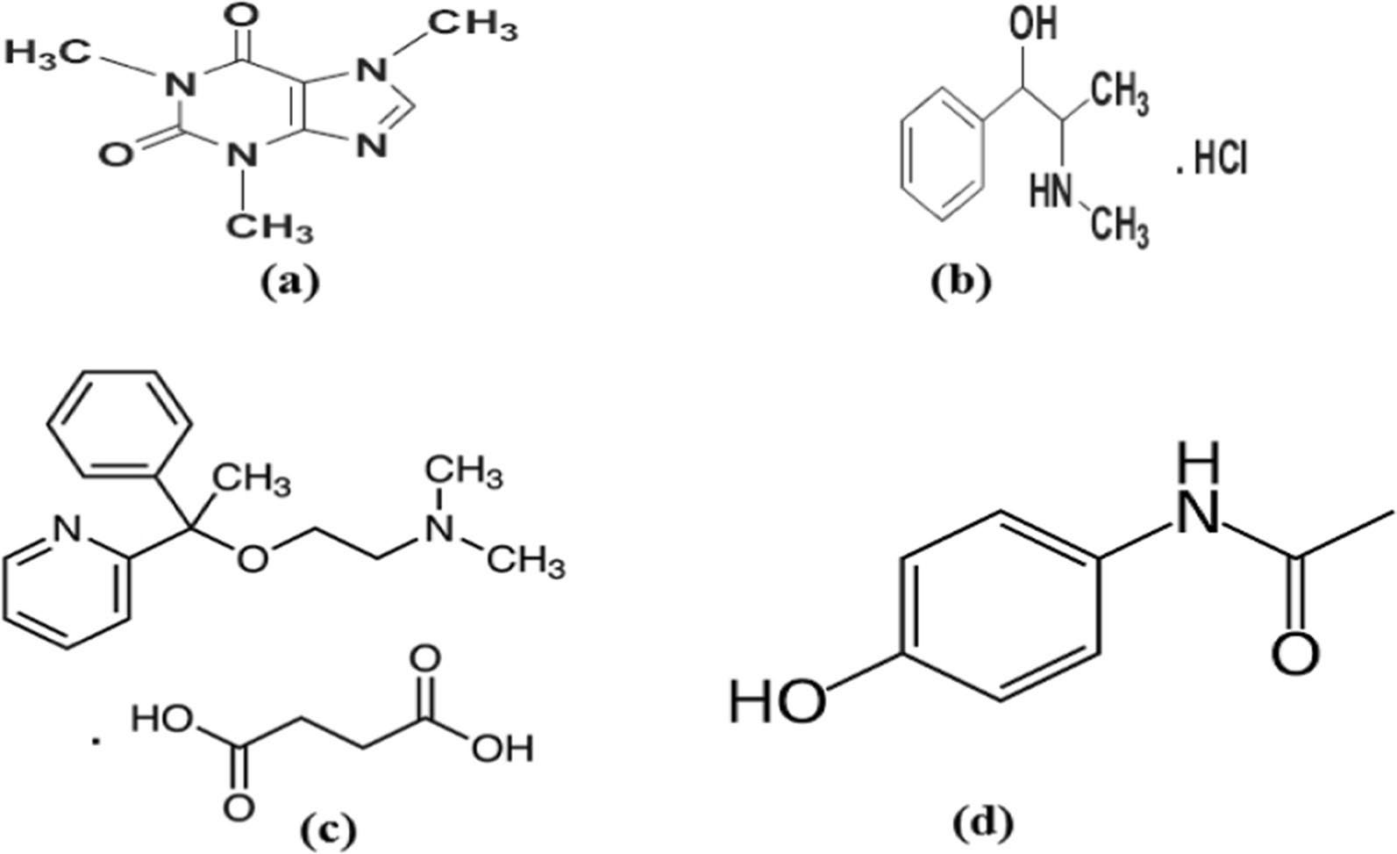
The chemical structure of (**a**) CAF, (**b**) PSE, (**c**) DOX, (**d**) PAR

Caffeine (CAF, Fig. 1a) is (1, 3, 7-trimethylxanthine). It can be present in coca nuts, coffee grounds, tea, and cocoa beans. Its pharmacological action is central nervous system stimulant, respiratory muscle relaxant, stimulate gastric acid secretion and diuretic [1]. It is also used in beverages as a flavoring agent. Many methods were reported for CAF assay such as spectrophotometric [2–8] and separation methods [7–16].

Pseudoephedrine hydrochloride (PSE) [(1S, 2S)-2-methylamino-1-phenylpropan-1-ol hydrochloride] shown in (Fig. 1b). It has sympathomimetic effect causing vasoconstriction and nasal airways congestion [17, 18]. CAF is a component of pharmaceuticals that include stimulants, painkillers, cold cures, weight-loss products, bronchial and cardiac stimulants, and also medications for the treatment of acne and other skin issues. PSE can be taken alone or in combination with other NSAIDs such as ibuprofen or aspirin, antihistamines, guaifenesin, dextromethorphan, and/or paracetamol. Several methods were used for its determination either alone or in combination with others including different spectrophotometric methods [19] and separation techniques [20–26], also by potentiometric method [27].

Doxylamine succinate (DOX); (Fig. 1c) is antihistaminic drug used for the treatment of allergy, hay fever, and common cold [28]. DOX is co-administered with Vitamin B6 (pyridoxine) to decrease morning sickness specialy during pregnancy [29]. Many spectrophotometric methods [30–33]and separation techniques [34–40] were reported for its analysis.

Paracetamol (PAR), (acetaminophen) is [N-(4-hydroxyphenyl) acetamide] (Fig. 1d). PAR has analgesic and antipyretic effect in numerous cold and flu remedies. Several methods are used for its assessment comprising: titrimetric [41], spectroscopic [42–46] and separation methods [47–50]. Many methods were reported for component with low absorbitivitiy [51], drug mixture with ratio variation and minor componants such as Fourier transform infrared spectroscopy (FTIR) combined with chemometric techniques [52].

To date, and to the best of our knowledge, no analytical methods are reported for the quantification of CAF, PSE, DOX and PAR, simultaneously, in their mixtures. These four drugs show severe spectra overlapping which hinders UV-spectrophotometric analysis in addition to the critical ratio in the pharmaceutical preparations and the presence of minor components. It is a challenge to resolve spectral overlapping of multi component mixtures without prior separation. However, in recent years, it has been proposed to use successive and progressive spectrophotometric resolution to examine ternary mixtures that partially or completely overlap where enrichment of a minor component in a combination may not affect the concentrations of major component(s) in higher ratio and reach their quantification limit.

In this study, in Lab sample enrichment technique was investigated for quantitative analysis of quaternion mixture by spiking the lowest concentration components with known concentration of its pure form. Cafamol extra tablets containing CAF, PSE, DOX and PAR in critical ratio (3:3:1:150) is widely used in the local market for relieving all symptoms in common cold.

In the present work, we describe selective, valid, sensitive method for the analysis of CAF, PSE and DOX, simultaneously as minor components as well as PAR (a major component) in their dosage form by applying sequential spectrophotometric resolution technique (SSRT) augmented with enrichment by spiking the lab samples with the lowest concentration components.

Other than a few co-formulated prescription drugs that are either poorly absorbed or present in small amounts and therefore are outside of their quantification limits. This technique is successfully applied to combined drugs in dosage forms containing challengeable ratios within the quantitative limit. When these components are greatly enhanced in samples, their combined spectrophotometric signals increase, which reduces the deviation from Beer's law that happens when a component's contribution is low [53]. Following mixture analysis, in lab-made mixtures, pure standards of the mild constituent(s) in known amounts are spiked. To increase the concentration of the minor components and yield a spectrum of augmented components with concentrations within the quantitative limit. The same techniques were used to assess the additional concentration. The linear regression equation of the suggested technique was used at the designated spectrum and the actual added concentrations of the proposed drug were determined. Finally, difference between enhanced and added ones were calculated. Also, the concentration of the stated drugs in the combination is determined.

Successive spectrophotometric resolution technique (SSRT) [54–56] depends on utilizing original mathematical techniques. So, the constants found in the spectral analysis were done to clarify spectral overlapping of multicomponent dose forms. Ratio subtraction has recently been employed as a resolution method where stepwise elimination was used to remove interference from one or more components in the mixture [57–60]. In this study, we used the SSRT to solve the challenges of overlapping spectra with low absorptivity and significant ratio variation. Following is an explanation of the theory:

We might separate out the components of W, X, Y, and Z using sequential ratio subtractions if Z renewed further than Y, Y was, in turn, more prolonged than X, and X is more extended than W. W was then established. Therefore, W might be determined by consecutive ratio subtraction using the mixture's spectrum as a divisor (Z') and a specific concentration of Z and the other components can be determined by the same way. This can be summarized as follows:

1 $$\left( {{\mathbf{W}} + {\mathbf{X}} + {\mathbf{X}} + {\mathbf{Z}}} \right)/{\mathbf{Z^{\prime}}} = \left( {{\mathbf{W}}/{\mathbf{Z^{\prime}}}} \right) + \left( {{\mathbf{X}}/{\mathbf{Z^{\prime}}}} \right) + \left( {{\mathbf{X}}/{\mathbf{Z^{\prime}}}} \right) + \left( {{\text{Z}}/{\mathbf{Z^{\prime}}}} \right)$$

2 $$\left( {{\mathbf{W}} + {\mathbf{X}} + {\mathbf{Y}} + {\mathbf{Z}}} \right)/{\mathbf{Z^{\prime}}} = \left( {{\mathbf{W}} + {\mathbf{X}} + {\mathbf{Y}}} \right)/{\mathbf{Z^{\prime}}} + {\mathbf{Constant}}$$

Directly from the (W + X + Y + Z)/Z' spectrum, the constant could be found by following the straight line that operated parallel to the wavelength axis in the region where Z was extended.

Constant multiplication method [58–60] had been devised as a new strategy for obtaining the first component (Z), in which Z could be calculated by multiplying Z' divisor by the previously obtained constant Z / Z', so we could obtain the D^o^ curve of Z. The following could serve as a summary:

3 $${\mathbf{Z}} = {\mathbf{Z}}/{\mathbf{Z^{\prime}}} \cdot {\mathbf{Z^{\prime}}}$$

The concentration of Z was calculated from the corresponding regression equation (obtained by plotting the absorbance values of the zero order curves of Z at its λ_max_ against the corresponding concentrations).

If we subtracted the measured value of the constant from the ratio spectrum Eq. (2), then multiplied the new spectrum by Z' divisor; we obtain the spectrum of W + X + Y as tertiary mixture.

This could be summarized in the following equations: **(*W* + *X* + *Y)/Z'. Z'* + constant–constant = *(W* + *X* + *Y)/Z'. Z'* = *W* + *X* + *Y.***then devise the new spectrum by Yʹ devisor

4 $$\left( {{\mathbf{W}} + {\mathbf{X}} + {\mathbf{Y}}} \right)/{\mathbf{Y^{\prime}}} = \left( {{\mathbf{W}}/{\mathbf{Y^{\prime}}}} \right) + \left( {{\mathbf{X}}/{\mathbf{Y^{\prime}}}} \right) + \left( {{\mathbf{Y}}/{\mathbf{Y^{\prime}}}} \right)$$

5 $$\left( {{\mathbf{W}} + {\mathbf{X}} + {\mathbf{Y}}} \right)/{\mathbf{Y^{\prime}}} = \left( {{\mathbf{W}} + {\mathbf{X}}} \right)/{\mathbf{Y^{\prime}}} + {\mathbf{Constant}}$$

By multiplying the constant by ***Y’*** devisor, compound Y will be obtained. Furthermore, It was noted that, the D^0^ curve of ***Y****.* This could be summarized as follows ***Y*** = ***Y****/****Y’****. ****Y’*** therefore we could obtain the D^0^ curve of Y. The concentration of Y was calculated from the corresponding regression equation (obtained by plotting the absorbance values of the zero order curves of Y at its λ_max_ against the corresponding concentration.

If we subtracted the measured value of the constant from the ratio spectrum Eq. (5), then multiplied the new spectrum by ***Y'****,* we obtain the spectrum of W + X as binary mixture. This could be summarized in the following equations:

6 $$\left( {{\mathbf{W}} + {\mathbf{X}}} \right)/{\mathbf{Y^{\prime}}} + \, {\mathbf{Constant}}{-}{\mathbf{Constant}}$$

7 $$\left( {{\mathbf{W}} + {\mathbf{X}}} \right)/{\mathbf{Y^{\prime}}} \cdot {\mathbf{Y^{\prime}}} = {\mathbf{W}} + {\mathbf{X}}$$

The obtained spectrum was successively divided by X as a divisor (Xʹ). The constant could be determined directly from the (W + X)/Xʹ spectrum by the straight line that was parallel to the wavelength axis in the region where X was extended.

8 $$\left( {{\mathbf{W}} + {\mathbf{X}}} \right)/{\mathbf{X^{\prime}}} = \left( {{\text{W}}/{\mathbf{X^{\prime}}}} \right) + \left( {{\mathbf{X}}/{\mathbf{X^{\prime}}}} \right)$$

For obtaining the third component (X), by multiplication of Xʹ divisor by the previously obtained constant X/ Xʹ, therefore we could obtain the D^0^ curve of X. This could be summarized as follows:

9 $${\mathbf{X}} = {\mathbf{X}}/{\mathbf{X^{\prime}}} \cdot {\mathbf{X^{\prime}}}$$

The concentration of X was calculated from the corresponding regression equation (obtained by plotting the absorbance values of the zero order curves of X at its λ_max_ against the corresponding concentrations). If we subtracted the measured value of the constant X / Xʹ from the ratio spectrum Eq. (8), then multiplied the spectrum by Xʹ, we obtain D^0^ curve of W. This could be summarized in the following equations:

10 $$\left( {{\mathbf{W}}/{\mathbf{X^{\prime}}}} \right) + {\mathbf{Constant}} - {\mathbf{Constant}} = {\mathbf{W}}/{\mathbf{X^{\prime}}}$$

11 $${\mathbf{W}}/{\mathbf{X^{\prime}}} \cdot {\mathbf{X^{\prime}}} = {\mathbf{W}}$$

The aim of this work was to utilize SSRT augmented with in-Lab enrichment technique as a robust method for simultaneous quantification of CAF, PSE, DOX and PAR in quaternary mixture.

## Experimental

### Apparatus

The measurments were done using UV- spectrophotometer (Jasco; Japan).

### Experimental materials

#### Samples

The studied authentic samples; CAF, PSE, DOX, PAR were Kindly obtained from Amriya-Company, Egypt, with purity of 99.6 ± 0.3 and 100.1 ± 0.08, for CAF and PSE according to USP and BP methods [9, 62] respectively. For DOX 100.4 ± 0.2 and 99.7 ± 0.13 for PAR assured by the reported ones [34, 48].

#### Finished product

Cafamol Extra® tablets that contain 30.0 mg of both CAF, PSE, 3.0 mg of DOX and 450.0 mg PAR were obtained from local market.

#### Solvents

Methanol, grade-A was the product of Honeywell, Germany.

### Standard solutions

To get (1000.0 µg/mL, stock solution), dissolving 100.0 mg of each CAF, PSE, DOX and PAR in 100.0 mL methanol.

## Procedure

### Spectral characteristics

Using methanol as a solvent, a series of standard solutions corresponding to 1.0–22.0 CAF, 1.0–24 PSE, 10.0–90.0 DOX, and 1.0–15.0 g/mL PAR were created from the stock standard solution. Then the UV range of the spectra was scanned.

### Calibration graphs

**Fig. 2:**
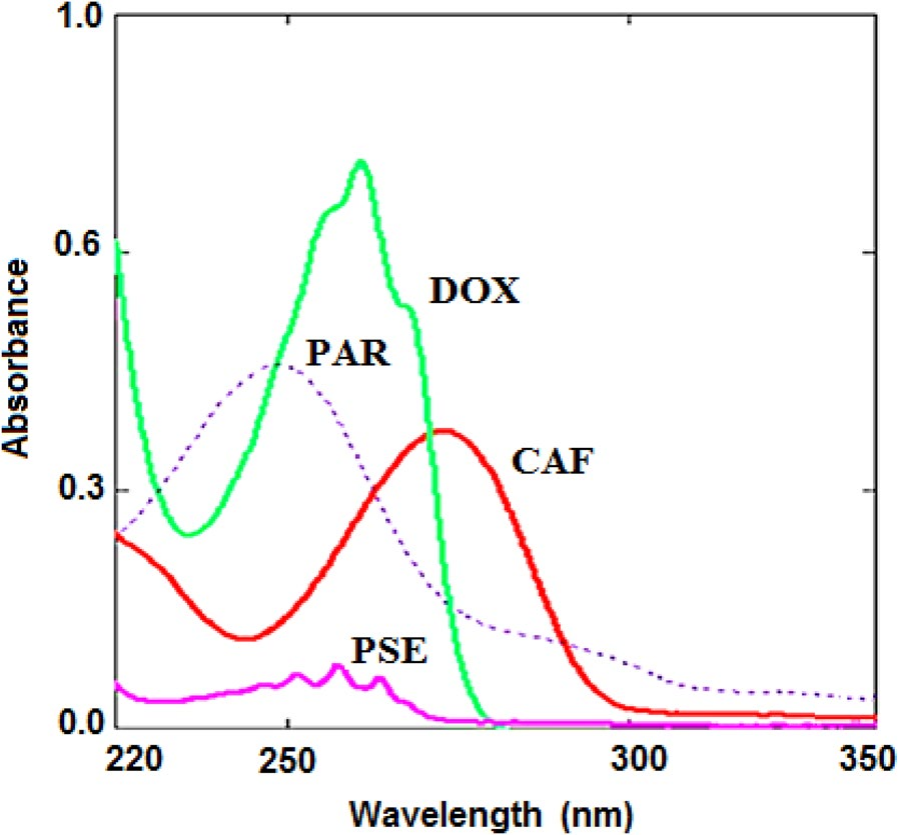
Zero order- spectra for a quaternion mixture of CAF, PSE, DOX and PAR (2.0, 22.0, 20.0, and 1.0 µg/mL), respectively

The qualified solution's peaks were collected as can be seen in (Fig. 2). The calibration graphs were made for the following; CAF, PSE, DOX, and PAR at 272.0, 257.0, 260.0 and 248.0 nm, respectively.

### Application of the proposed method in lab-mixtures

**Fig. 3:**
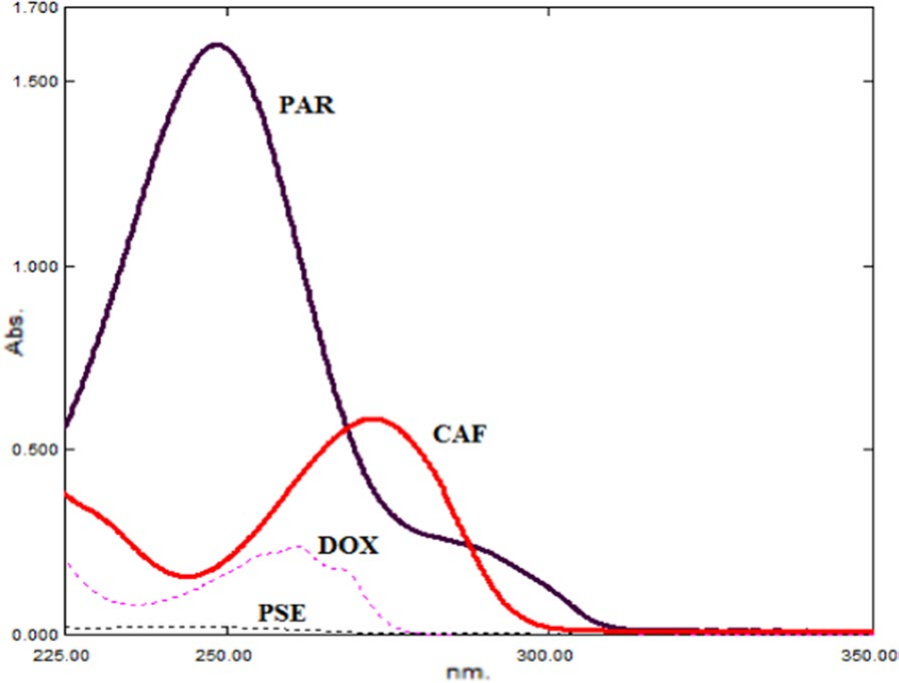
Zero order- spectra for a quaternion mixture of CAF, PSE, DOX, and PAR (11.0, 20.1, 11.0, and 15.0 µg/mL), respectively after enrichment

In a series of 10.0-mL volumetric flasks, serial dilutions of CAF, PSE, DOX, and PAR were precisely transferred from their working solutions, thoroughly mixed, and then each flask was enriched with (10.0, 10.0, and 20.0 µg/mL) of CAF, PSE, and DOX, respectively, and then filled to the mark volume with methanol. This procedure was done in order to create mixtures containing various ratios of the studied drugs. The spectra of this mixtures were scanned in range 200 to 400 nm as shown in (Fig. 3).

**Fig. 4:**
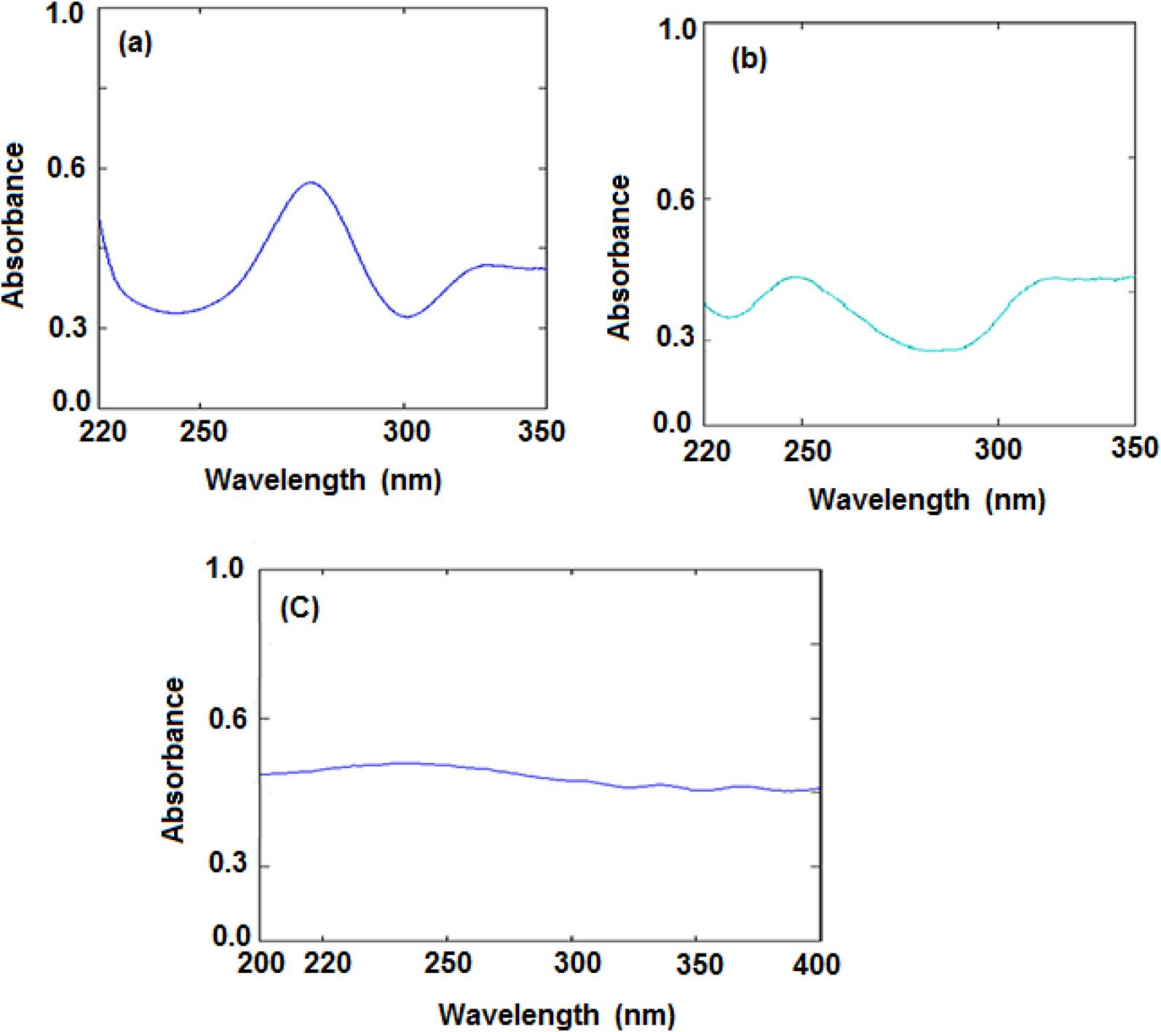
Division spectra of lab-mixtures; (**a**) PAR (15 µg/mL), (**b**) CAF (20 µg/mL), and (**c**) DOX (50 µg/mL) as divisors, respectively

Afterward, these spectra were successively divided by the PAR spectrum at a concentration of 15.0 µg/mL, and the plateau area at (319.0–335.0 nm) was used to measure the amplitudes of the PAR/ PAR' (I) constants and subtract them (Fig. 4a).

The constant amplitudes were collected at (278.0–290.0 nm) region, preceded by multiplying of the spectrum of CAF (20.0 µg/mL).

The collected peaks were divided using standard DOX (50.0 µg/mL) as a divisor to determine the amplitudes of the constant of DOX/DOXʹ (III) (Fig. 4c). The amplitudes of the constants were measured at (262.0–270.0 nm) region, then multiplied by the spectrum of the standard DOX (50.0 µg/mL) to obtain the PSE spectrum. The proposed drugs' zero order absorption spectra could be obtained by multiplying the values (I, II, III) by the zero order absorption spectra of standard PAR (15.0 µg/mL), CAF (20.0 µg/mL), and DOX (50.0 µg/mL), respectively.

The proposed method's matching regression equation at the appropriate wavelength was used to estimate the actual added and augmented concentrations of the cited drug(s).

### Application to the finished product

A quantity precisely equal to 30.0 mg of CAF, PSE, 3.0 mg of DOX, and 450.0 mg of PAR was weighed from ten Cafamol extra® tablets, finely powdered, transferred into beaker (100.0 mL). Afterthat 30.0 mL of the selected solvent (methanol) was used, ultrasonically processed for 15 min, and then filtered into a 100.0 mL volumetric flask. The solution was then finished to the mark with methanol. Next, wash the residue three times with 15.0 mL of methanol each time. To prepare a solution with 11.0 mg/mL of CAF, PSE, 20.1 mg/mL of DOX, and 15.0 mg/mL of PAR, accurately transfer an aliquot into a 10.0-mL calibrated flask. Next, spike the solution with standard solutions of 10.0, 10.0, and 20.0 mg/mL of CAF, PSE, and DOX from each of their stock solutions, respectively, and adding methanol to reach the desired volume. The method described under "Analysis of laboratory prepared mixtures" was used to determine each drug's dosage form as a tablet. Prior to using the previously mentioned methods, the dosage form was mixed with various known concentrations of pure standard CAF, PSE, DOX, and PAR using the standard addition technique. After calculating the drug concentrations, it was possible to successfully calculate the mean recoveries.

## Results and discussion

Fastness and sensitivity are the main characters of spectrophotometric techniques. In this work we focused on applying a mathematical technique upon developing this method**.** The ratio of CAF, PSE, DOX and PAR in Cafamol extra tablets is critical ratio (3:3:1:150). To attain this ratio in their dosage form content, low CAF, PSE, and DOX concentrations above the range were required. simultaneous quantification of each drug concentration in zero order spectra was therefore very challenging; the enrichment technique (addition of the standers of minor components to their lab mixture) was therefore applied.

Each drug's absorptivity and spectral characteristics made it difficult to obey Beer's law. The dosage form ratios of CAF, PSE, DOX, and PAR in methanol were separately scanned and overlaid. According to Fig. 2, the spectra (zero ordering) of pure CAF, PSE, DOX, and PAR had a significant amount of overlap, making it difficult to determine all four simultaneously using standard spectrophotometric techniques. In the UV region, PAR was longer than the other components (200.0–400.0 nm). Also, CAF was more extended than DOX which is more extended than PSE.

It is obvious from previously reported methods for analysis of mixtures that there is no spectrophotometric method offered an opportunity to enable the quaternion mixture of the studied drugs to be analysed simultaneously, simply, accurately, precisely and economicaly. In the present study the developing lab-samples enrichment technique and successive ratio subtraction (SRS) spectrophotometric method were able to evaluate these drugs by resolving the overlapping spectra and low absorptivities and gap ratio, without previously separation steps. Also there no need to determine the standard added as the mathematical factorization (divition and subtraction) extract the D^0^ curves, in addition that the absorbitivities and/or dervatization ratios are not required.

### Successive ratio subtraction coupled with constant multiplication (SRS–CM)

**Fig. 5:**
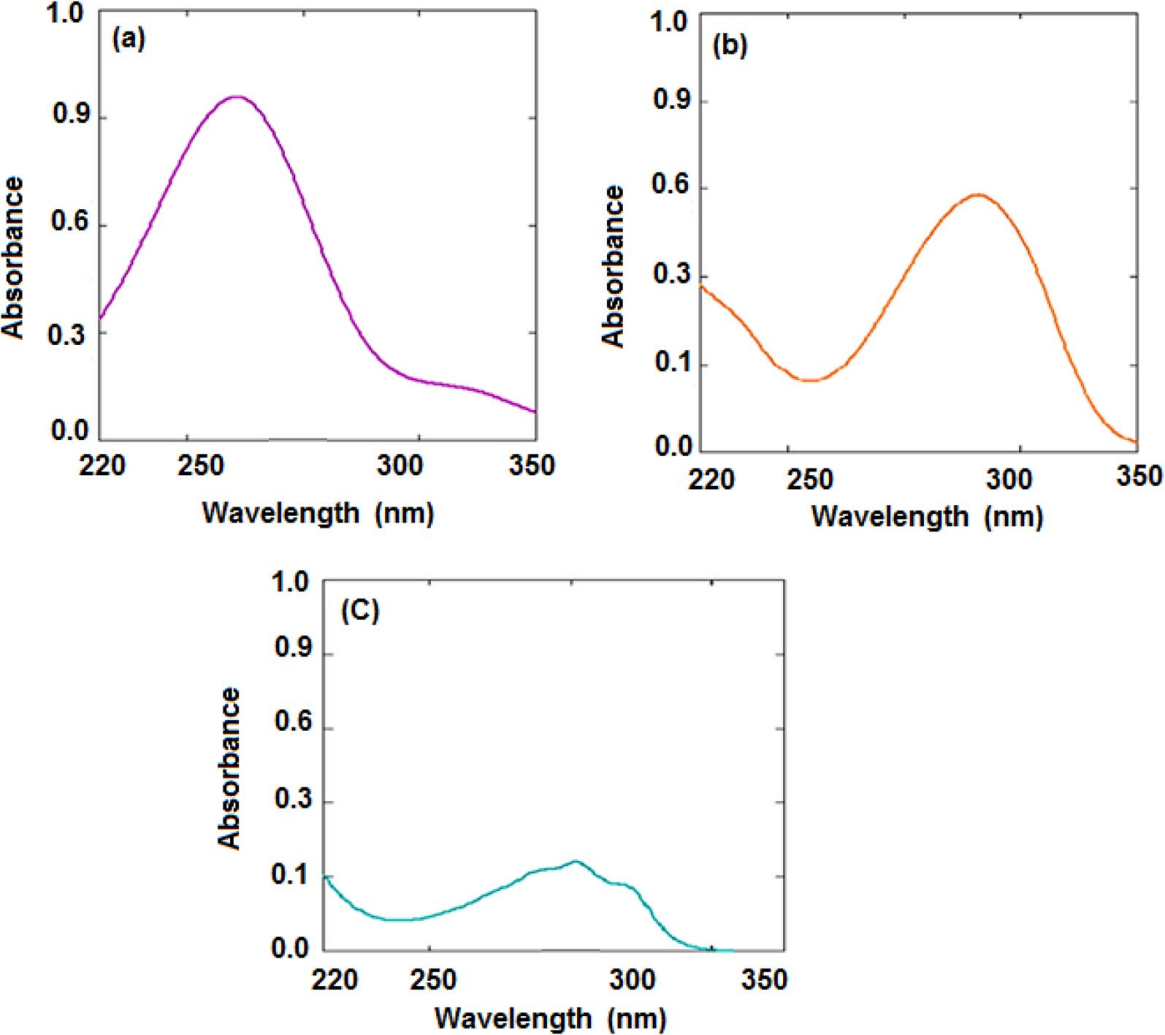
Zero-order spectra of (**a**) PAR (15.0 µg/mL), (**b**) CAF (11.0 µg/mL), and (**c**) DOX (20.1 µg/mL)

The PAR was longer than CAF, which was longer than DOX, which was longer than PSE, according to the zero-order absorption spectrum (D^0^) (Fig. 2). Researchers were able to calculate a constant from the straight line parallel to the wavelength axis at the extended part (PAR/PAR') by dividing the spectrum of the lab-mixture by a divisor spectrum of standard 15.0 µg/mL PAR at (319.0–335.0 nm) as showed in Fig. 4a. This constant was multiplied by the divisor spectrum (PAR') to produce the original PAR curve, and the absorbance at its maximum wavelength of 248.0 nm also was quantified (Fig. 5a).

A plateau region parallel to the wavelength axis was apparent in the extended range of the acquired division spectrum, and this allowed us to determine the constant (CAF/CAF') (278.0–290.0 nm) (Fig. 4b). This constant was multiplied by the (CAF') to create the original CAF curve (Fig. 5b), from which the absorbance at its maximum wavelength of 272.0 nm was determined. Their regression equations represented the linear relationship between the absorbance at 272.0 nm and the CAF concentrations, and the CAF concentration was determined by substituting them.

This constant (CAF/CAF') was subtracted to yield the CAF D^0^ spectrum. D0 spectra of DOX and PSE were obtained by dividing this result by the spectrum of the divisor. It was feasible to obtain the longer D0 spectrum of DOX by using the regular DOXʹ (50.0 µg/mL) D^0^ spectrum as a divisor. The ensuing division spectrum showed a plateau region at the expanded region parallel to the wavelength axis, from which the constant DOX/DOXʹ (262.0–270.0 nm) could be derived (Fig. 4c).

**Fig. 6:**
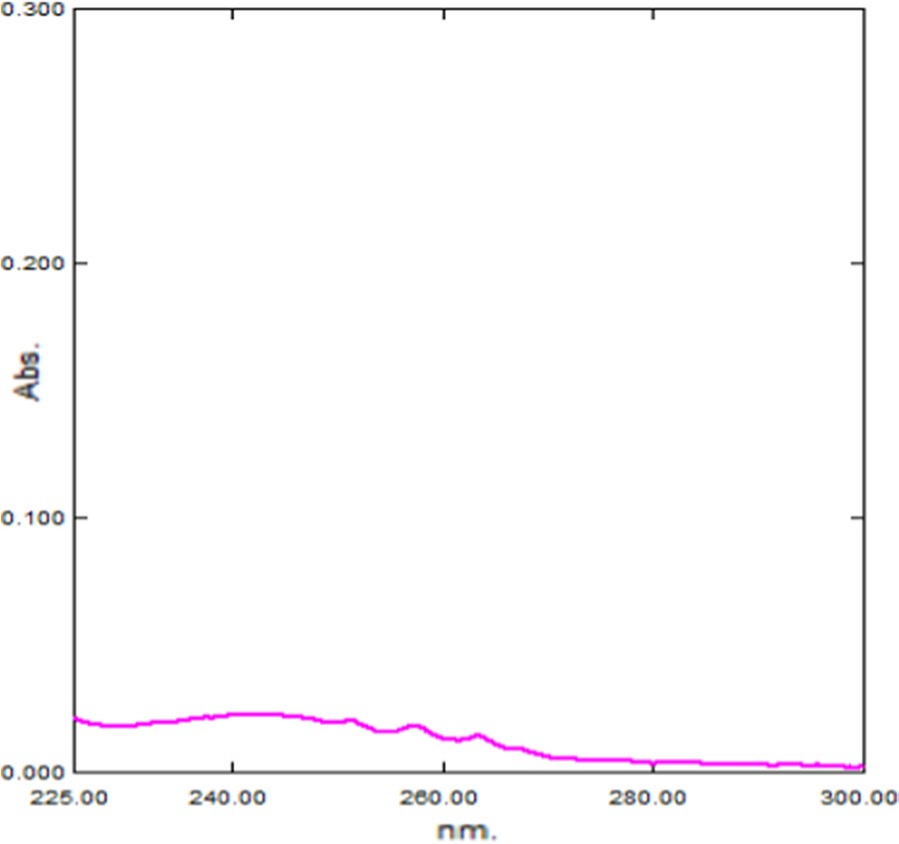
Zero-order spectra of PSE (11.0 µg/mL) after subtraction and multiplication by a divisor DOX (50.0 µg/mL)

This constant was multiplied by (DOXʹ) to get the DOX original curve (Fig. 5c), from which the absorbance at its maximum wavelength of 260.0 nm was calculated. The concentration of DOX was determined by swapping the linear relation between both the concentrations of DOX and the absorbance at the maximum wavelength, 260.0 nm, in their linear regression. Zero-order spectrum of the initial PSE curve was calculated (Fig. 6). Then, using zero order absorption spectra at its maximum wavelength of 257.0 nm, measuring the absorbance.

### Method optimization

It's essential to optimize the numerous variables that may have an impact on the method.

#### Effect of solvent

Different solvents were tested like; acetonitrile, distilled water, and methanol.It was noted that methanol was the suitable solvent for dissolving the examined drugs.

#### The divisor and its concentration

To find a balance between the choice of the divisor concentration was essential for achieving the good sensitivity and the least amount of noise. As divisors for the quantification of the cited drugs in their pure form and/or laboratory-prepared mixtures, various concentrations of PAR (15.0, 10.0 and 5.0 µg/mL), CAF (20.0, 14.0 and 8.0 µg/mL), and DOX (70.0, 50.0 and 40.0 µg/mL) were tested.

#### Optimization of the constants

It was found that the best divisors that give the highest sensitivity, recovaries, minimum noise and repeatability are 15.0, 20.0, and 50.0 µg/mL for PAR, CAF and DOX, respectively.

## Method validation

The validity of the sugggested analytical methods was prformed based on ICH/Q2 rules [61].

### Linearity

A series of standard solutions were used to get calibration graphs and the linearity’s were achieved as presented in Table 1.

**Table 1:** Different validated parameters of the present method

Parameters	CAF	PSE	DOX	PAR
Linearity range (µg/mL)	1.0–22.0	1.0–24.0	10.0–90.0	1.0–15.0
Slope	0.0528	0.0423	0.0125	0.1015
Intercept	0.0052	0.0918	0.0031	0.0809
LOD (µg/mL)	0.021	0.124	0.186	0.137
LOQ (µg/mL)	0.070	0.414	0.621	0.456
Correlation coefficient (*r*)	0.9999	0.9997	0.9998	0.9997
Accuracy (%R)^a^				
Mean ± SD	100.71 ± 0.851	99.21 ± 1.114	100.5 ± 0.772	99.88 ± 1.020
Precision (%RSD)^b^				
Repeatability	0. 938	0.911	0.613	0.508
Intermediate precision	0.953	0.917	0.745	0.553

^a^Nine determinations average

^b^Nine determinations precision

### Precision

Two level (intra and inter-day) precision were calculated by processing the various calibration graphs over the course of three different days, the proposed method's linearity was assessed; Small values % RSD, guaranteed elevated method precision; the outcomes are shown in Table 1.

### Accuracy

Acceptable recovery percentage (%R) and SD-values ensure the method is accurate are summarized in Tables 1, 2. The results obtained suggested that the developed methods were accurate.

**Table 2:** Assay of CAF, PSE, DOX and PAR in lab-mixtures by the proposed analytical method

Lab mixture	Concentration (µg/mL)	In lab recovery*			
		CAF	PSE	DOX	PAR
A	11:11:20.1:15	100.02	99.98	98.23	100.54
B	12:12:21:10	100.68	99.58	98.68	101.01
C	14:14:22:8	99.21	101.52	100.23	100.35
D	16:16:23:6	98.87	100.04	100.12	98.78
Mean ± SD		99.70 ± 0.815	100.28 ± 0.85	99.32 ± 1.011	100.17 ± 0.967

*Each experiment repeated three times

### Detection and quantification limits; (LOQ &LOD)

Table 1 showed the resulted data of LOD and LOQ calculation.

### Selectivity

Selectivity was evaluated at various mixtures that contained the investigated drugs in a variety of different ratios. The resulted data (% RSD and % recovery) were summarized in Table 3.

**Table 3:** Standard addition technique of CAF, PSE, DOX and PAR in Cafamol Extra® tablets

Sample	CAF	PSE	DOX	PAR
Cafamol extra ® tablets (Found% ± S.D)*	98.21 ± 0.954	100.82 ± 0.0.748	99.22 ± 0.369	98.66 ± 0.528
Standard addition (Recovery % ± SD)**	100.32 ± 0.901	99.34 ± 0.712	99.64 ± 0.321	98.47 ± 0.257

*Each experiment repeated three times of three concentration of the tablets (20,40,60 mg)

**Means of nine determinations

### Statistical analysis

For the CAF, PSE [9, 62], and PAR [48] as well as the published method [34] for DOX, Comparative statistics were displayed in Table 4 between the findings data using suggested method and those that were acquired via approved methods. The calculated "t" and "F" values were lower than the theoretical values, demonstrating that there was no real difference between the accuracy and precision of the suggested protocol and those of the official or reported methods.

**Table 4:** Comparative study between the proposed and reported methods for CAF, PSE, DOX and PAR in their pure forms

	CAF		PSE		DOX		PAR	
	SSRT	*Official method [9]	SSRT	**Official method [62]	S,SRT	Reported method [34]	SSRT	Reported method [48]
Mean	100.26	100.02	100.19	100.33	100.03	99.74	100.31	99.98
SD	0.31	0.53	0.288	0.55	0.6	0.69	0.42	0.25
Variance	0.096	0.281	0.083	0.303	0.360	0.482	0.176	0.063
N	6	5	6	5	6	5	6	5
Student’s t-test (1.833)	0.893		0.514		0.757		1.612	
F value (6.256)	2.923		3.647		1.323		0.354	

P = 0.05 Numbers between brackets are the tabulated ones

*Official RP-HPLC method for determination of CAF using mobile phase, acetonitrile: sodium acetate buffer (45:55 v/v) pumped at a flow rate of 1.0 mL/min through the column (C18; 150.0 mm × 4.6 mm, 5.0 mm). **Official RP-HPLC method for determination of PSE using mobile phase, Methanol: Triethylamine phosphoric acid solution (pH 6.8) (10:90 v/v) pumped at a flow rate of 0.6 mL/min through the column (C18; 150.0 mm × 3 mm, 5.0 mm) at 30 _C. ***Direct UV spectrophotometric method, measuring the absorbance in water at 244 nm.

## Conclusion

The proposed method utilizes SSRT augmented with in-lab enrichment techniques as a robust method for simultaneous quantification of CAF, PSE, DOX, and PAR in a quaternary mixture. This augmentation allows the determination of any mixtures containing components in critical ratios. These materials are included in samples to increase their concentrations, which improve their individual spectrophotometric signals and permit dedication. The recovery and reproducibility of the analysis results with an actual tablet sample, as well as a comparison between the mean contents of active substances in the tablets obtained using the suggested method and those obtained using the official analytical methods for CAF, PSE, and PAR, as well as the reported method for DOX, were used to determine the procedure's reliability.

## Data Availability

All data generated or analyzed during this study are included in this published articles.

## Abbreviations

CAF: Caffeine
CM: Constant multiplication
D^0^: Zero-order absorption spectrum
DOX: Doxylamine succinate
FTIR: Fourier transform infrared spectroscopy
ICH: International Conference on Harmonization
LOD: Limits of detection
LOQ: Limits of quantification
PAR: Paracetamol
PSE: Pseudoephedrine
RSD: Residual standard deviation
SSRT: Sequential spectrophotometric resolution technique
SRS: Successive ratio subtraction
SRS–CM: Successive ratio subtraction coupled with constant multiplication
